# Charge Configuration Memory Devices: Energy Efficiency and Switching Speed

**DOI:** 10.1021/acs.nanolett.2c01116

**Published:** 2022-06-10

**Authors:** Anze Mraz, Rok Venturini, Damjan Svetin, Vitomir Sever, Ian Aleksander Mihailovic, Igor Vaskivskyi, Bojan Ambrozic, Goran Dražić, Maria D’Antuono, Daniela Stornaiuolo, Francesco Tafuri, Dimitrios Kazazis, Jan Ravnik, Yasin Ekinci, Dragan Mihailovic

**Affiliations:** † Complex Matter Department F7, Jozef Stefan Institute, Jamova cesta 39, 1000 Ljubljana, Slovenia; ‡ CENN Nanocenter, Jamova cesta 39, 1000 Ljubljana, Slovenia; § Faculty of Mathematics and Physics, University of Ljubljana, Jadranska cesta 19, 1000 Ljubljana, Slovenia; ∥ Faculty of Electrical Engineering, University of Ljubljana, Tržaška cesta 25, 1000 Ljubljana, Slovenia; ⊥ Paul Scherrer Institute, Forschungsstrasse 111, 5232 Villigen PSI, Switzerland; # Dipartimento di Fisica “Ettore Pancini”, Università di Napoli Federico II, Monte S. Angelo via Cinthia, 80126 Napoli, Italy; ○ CNR-SPIN, Complesso Monte Sant’Angelo, Via Cinthia, 80126 Napoli, Italy; ● CNR-Istituto Nazionale di Ottica (CNR-INO), Largo Enrico Fermi 6, 50125 Florence, Italy; ∇ Jozef Stefan International Postgraduate School, Jamova cesta 39, 1000 Ljubljana, Slovenia; ∞ Department of Materials Chemistry, National Institute of Chemistry, Hajdrihova 19, 1000 Ljubljana, Slovenia

**Keywords:** charge configuration memory, TaS_2_, ultrafast, energy-efficient, cryogenic, nonvolatile

## Abstract

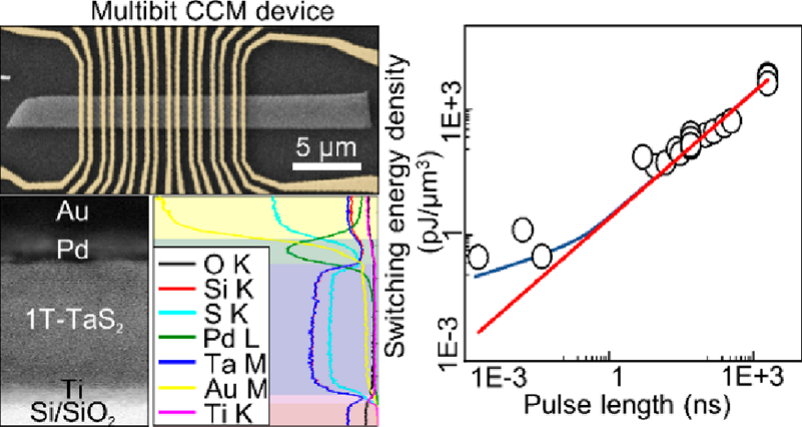

Current trends in data processing have given impetus for an intense search of new concepts of memory devices with emphasis on efficiency, speed, and scalability. A promising new approach to memory storage is based on resistance switching between charge-ordered domain states in the layered dichalcogenide 1T-TaS_2_. Here we investigate the energy efficiency scaling of such charge configuration memory (CCM) devices as a function of device size and data write time τ_W_ as well as other parameters that have bearing on efficient device operation. We find that switching energy efficiency scales approximately linearly with both quantities over multiple decades, departing from linearity only when τ_W_ approaches the ∼0.5 ps intrinsic switching limit. Compared to current state of the art memory devices, CCM devices are found to be much faster and significantly more energy efficient, demonstrated here with two-terminal switching using 2.2 fJ, 16 ps electrical pulses.

Research in the area of novel memory devices has been intense in the recent decades, but there have been few breakthroughs that have led to implementation.^1−3^ As a result, technologies such as cryocomputing that promise large improvements in energy consumption have been seriously hindered by the absence of a suitable fast and energy-efficient memory for more than two decades.^4,5^ As an alternative to modern magnetic devices, manipulating charge, rather than spins, for data storage could be more efficient because it can be directly driven by electrical charge injection that promises to be extremely fast and efficient. Unfortunately, the direct coupling of an electronic two-level system to lattice degrees of freedom causes rapid state decoherence and dissipation, which fundamentally limits charge-storage based memory concepts. As an apparent solution to this problem, it was shown recently that topological protection that stabilizes different charge density wave (CDW) domain states in the quasi-2D layered transition metal chalcogenide 1T-TaS_2_ can be used for memory devices.^6,7^ The basic mechanism for such a charge configuration memory (CCM) was shown to be unique to this material,^7−9^ involving reversible charge reconfiguration from an insulating, spatially uniform CDW state to a metallic domain state^10^ with accompanying restacking of the CDW along the direction perpendicular to the layers.^11^ To develop the CCM concept into viable practical devices, a better understanding of the device characteristics is required, particularly the operational limitations, such as write speed, energy efficiency, and endurance, as well as size scaling limitations and contact material compatibility. For low temperature operation, minimization of dissipation is essential, so investigation of how the switching energy scales with device size is particularly important. The write (W) cycle of the CCM device was shown to be nonthermal, while the erase (E) cycle is at least partially thermal.^7^ Intrinsic nonohmic behavior and dissipation at the contacts,^12^ especially if energy barriers are formed as a result of interface chemistry, may limit the energy efficiency in small devices.^13^ In this paper we investigate CCM scaling properties in the nonvolatile resistance switching region, which is particularly relevant for incorporation into cryocomputing environments where the device has potential applications. We investigate the electrical contact structure with high resolution electron microscopy, searching for possible interfacial layers that may limit device performance. We conclude by comparing the measured characteristics of CCM with other current and emerging memory devices.

The devices were nanofabricated by evaporating metal contacts on 1T-TaS_2_ single crystal flakes as can be seen on a scanning electron microscope (SEM) image in Figure 1a (see Methods in Suppporting Information). Switching characteristics were measured on many devices with very different gap sizes *L* (Figure 1b) between the Au electrodes, using a large range of “write” pulse lengths τ_W_, from 16 ps to 600 ms. For short pulse switching, low-attenuation transmission line circuits were used in combination with picosecond digital electronics (see Methods in Supporting Information). We first present an analysis of the contact structure in Au/1T-TaS_2_ devices on Si/SiO_2_ substrates. A bright-field scanning transmission electron microscope (BF-STEM) image of a cross-section of two electrodes (one memory bit) and a zoom-in on the interface between the metal electrodes and 1T-TaS_2_ can be seen in Figure 1b and Figure 1c, respectively. The layered structure of the 1T-TaS_2_ crystal is clearly visible in Figure 1c. Part of the cross-section showing the entire structure of the device can be seen in Figure 1d. An energy-dispersive X-ray spectroscopy (EDS) analysis of the device’s interfaces is presented in Figure 1e, along the line scan marked with the red line in Figure 1d. Considering the overlap in the EDS spectrum for certain characteristic lines of elements (Ta M and Si K, Au M and S K), the individual layers of the fabricated CCM device can be identified (Figure 1e). Importantly, multiple EDS analyses (at ∼1 nm resolution) on different devices do not show any evidence of an oxide layer at the interface between the 1T-TaS_2_ crystal and the metal electrode, consistent with a previous report^13^ and the voltage–current characteristics discussed below.

**Figure 1:**
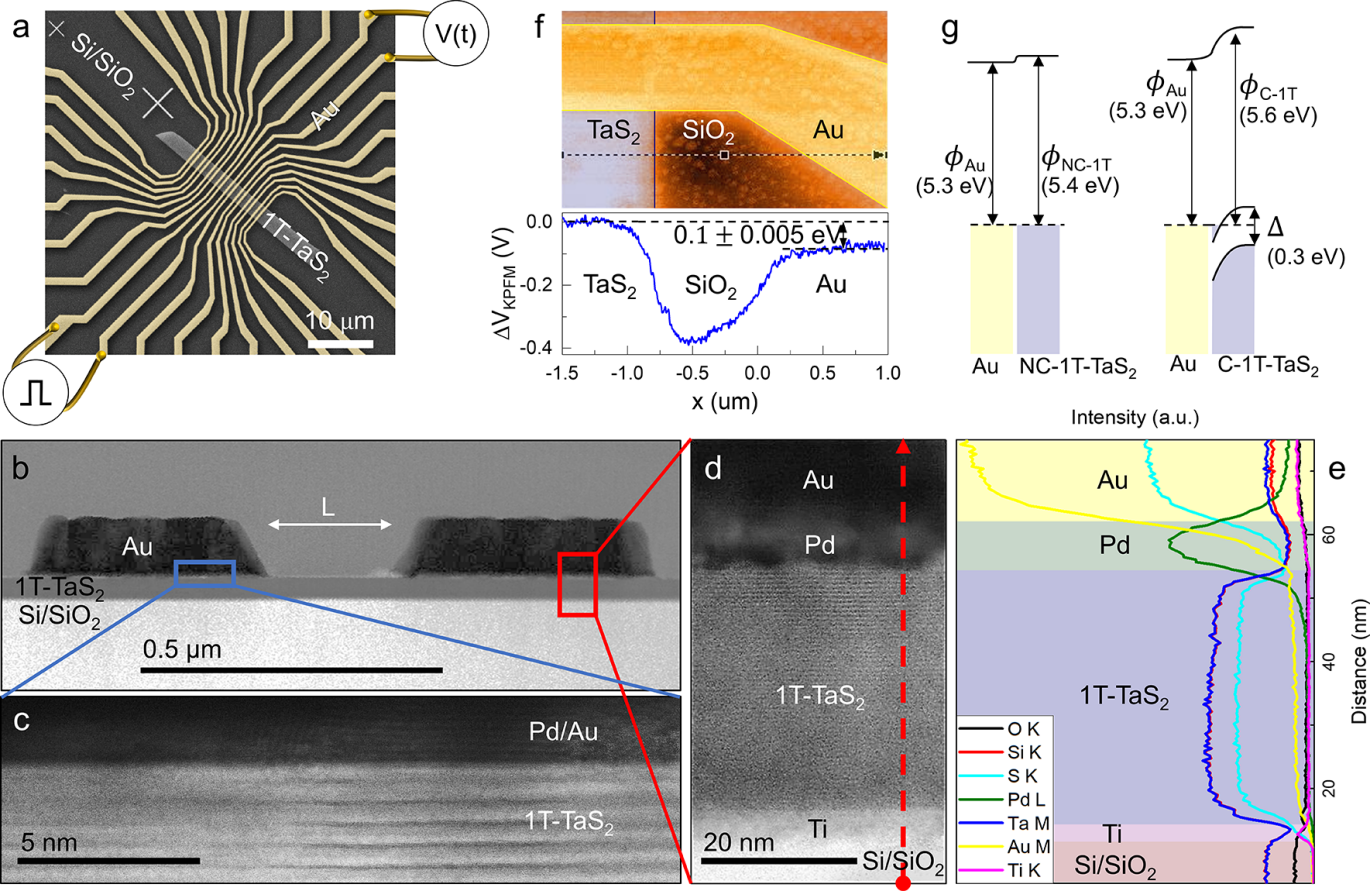
CCM device, EDS, and work function analysis. (a) SEM image of a typical CCM device used for measurements with transmission line contacts. (b) BF-STEM image of a cross-section of a fabricated CCM device (one memory bit). (c) Zoom-in to the interface between the metal electrode and 1T-TaS_2_ crystal. (d) Zoom-in to a part of the cross-section with the EDS line scan marked. (e) EDS analysis of the zoomed-in section, where individual layers are identified: Au (yellow), Pd (green), Ta (dark blue), S (light blue), Si (red), O (black), Ti (pink). (f) Top panel shows an AFM image of a part of the CCM device with the line scan for the KPFM measurement marked. Bottom panel shows KPFM measurement of the work functions for 1T-TaS_2_, SiO_2_, and Au electrodes along the line scan. (g) Schematic band diagram of the device based on the KPFM measurements. On the left is a band diagram of an interface between the Au electrode and the nearly commensurate (NC) metallic state at room temperature, and on the right is an interface between the Au electrode and the commensurate (C) charge density wave state at cryogenic temperature.

To further ascertain the possible role of contacts in device functionality we measured the relative difference in work functions  of the 1T-TaS_2_ and Au in a CCM device using a Kelvin probe force microscopy (KPFM). The upper panel in Figure 1f shows an atomic force microscope (AFM) image of a part of the CCM device, while the bottom panel in Figure 1f shows the KPFM potential difference  as a function of the scanning position on the device. The scanning is performed across the 1T-TaS_2_ crystal, the Si/SiO_2_ substrate and the Au electrode, as indicated with the dashed line in Figure 1f, top panel. While the absolute value of  was not calibrated, the relative difference of work functions is accurately determined,
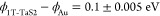
in agreement with published values for  = 5.5 ± 0.01 eV (single crystal)^14^ and  = 5.1–5.47 eV.^15^ Judging from the KPFM measurement and the values from ref (15), it looks like our gold layer has facets with predominantly (100) or (110) orientation. The corresponding band diagrams for the room-temperature nearly commensurate (NC) metallic state and the low-temperature commensurate charge density wave (C-CDW) are shown in Figure 1g. Ideally electron injection from Au metal into the 1T-TaS_2_ CDW-gapped semiconductor with a gap  eV ^16^ takes place (Figure 1g), and the junction is expected to be ohmic. 1T-TaS_2_ reacts to charge injection by forming a conducting interface layer parallel to the interface. We thus do not expect a barrier to form at the interface with Au contacts due to carrier diffusion, which is consistent with the observed linear voltage–current curves^6^ and the scaling behavior with device size and τ_W_ described in the following section.

While basic memristive switching of the CCM device at higher temperatures was discussed elsewhere,^6^ a measurement that demonstrates millikelvin operation and switching from the high resistance (HI) state  to low (LO) resistance state  is illustrated by the *R*–*T* curve in Figure 2a, for a device with intercontact distance  nm. The entire measurement cycle is shown starting from 280 K, cooling and switching at 350 mK by a single write (W) pulse, and heating back to 280 K, where the system reverts to its original state. Upon heating we observe characteristic steps, previously attributed to relaxation of domains.^7,17^ The insert to Figure 2a shows the *R*–*T* curves on an expanded scale, showing a commonly observed hysteresis associated with the presence of additional phases^18,19^ above 100 K.

**Figure 2:**
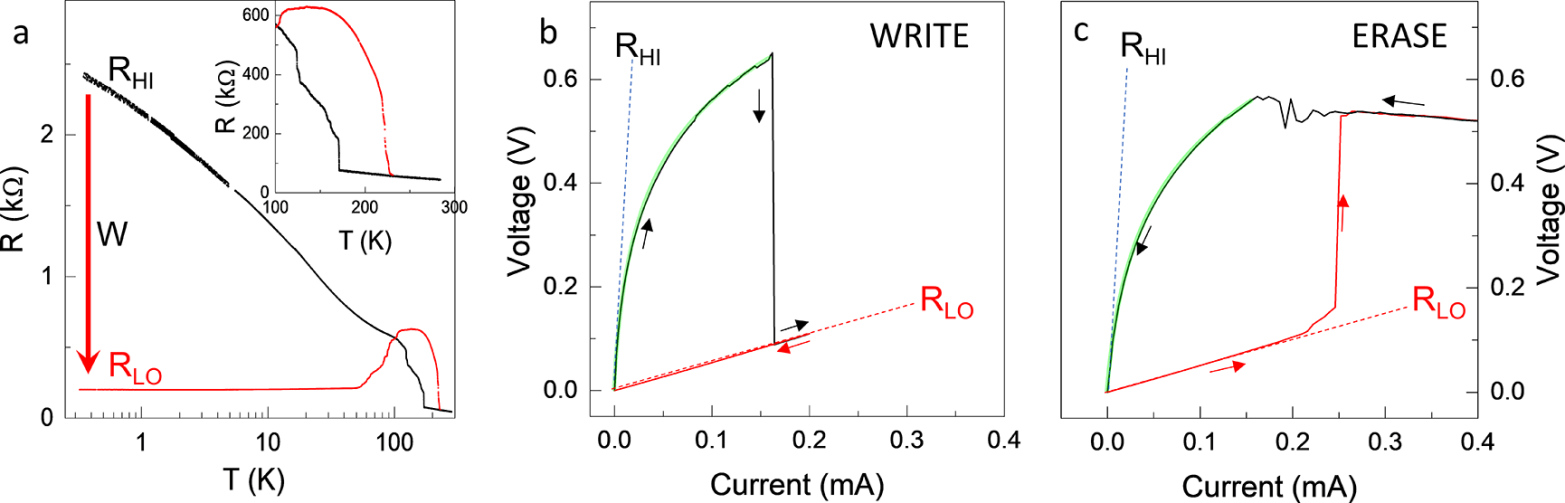
Resistance switching and voltage–current characteristics of CCM devices. (a) Temperature dependence of the four-contact resistance *R*. Switching from *R*_HI_ to *R*_LO_ at 350 mK is caused by an electrical W pulse as indicated by the arrow. Heating above 90 K (red line) reverts the system to the *R*_HI_ state. Inset to (a) shows the expanded scale of the *R*–*T* curve. (b, c) Pulsed measurements of the *V*–*I* curve for the W and E operations, respectively.

A typical voltage–current (*V*–*I*) curve for a W cycle at 20 K is shown in Figure 2b for *incrementally* increasing current pulses  through the CCM device with an intercontact distance  nm (see insert to Figure 3b). In this measurement, the pulses were very long (), illustrating the versatility of the device (fast measurements are shown later). The *V*–*I* measurement allows us to accurately determine the switching threshold for both the write and erase processes. We observe a nonlinear *V*–*I* curve with an initial slope  (dashed  line) up to the  mA. Above this current the voltage drops from 0.65 to 0.08 V and the device switches to a linear *V*–*I* relation with resistance  (dashed  line), remaining in this state indefinitely at this temperature. The *V*–*I* curve for W can be fit with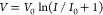 for  (green line) and  for  (dashed  line) in agreement with ref (6). To switch back to the pristine state, the erase (E) sequence is illustrated in Figure 2c where the current is ramped from  to 0.4 mA. Initially we follow the *R*_LO_ curve. Above a threshold  mA the device switches to a high resistance state. As current is lowered to zero, the device remains in  at low currents. The behavior is consistent with the previous *V*–*I* measurements with 10 μs pulses,^6^ implying that it is not dependent on the pulse length over the range τ_W_ = 10^–5^–1 s.

**Figure 3:**
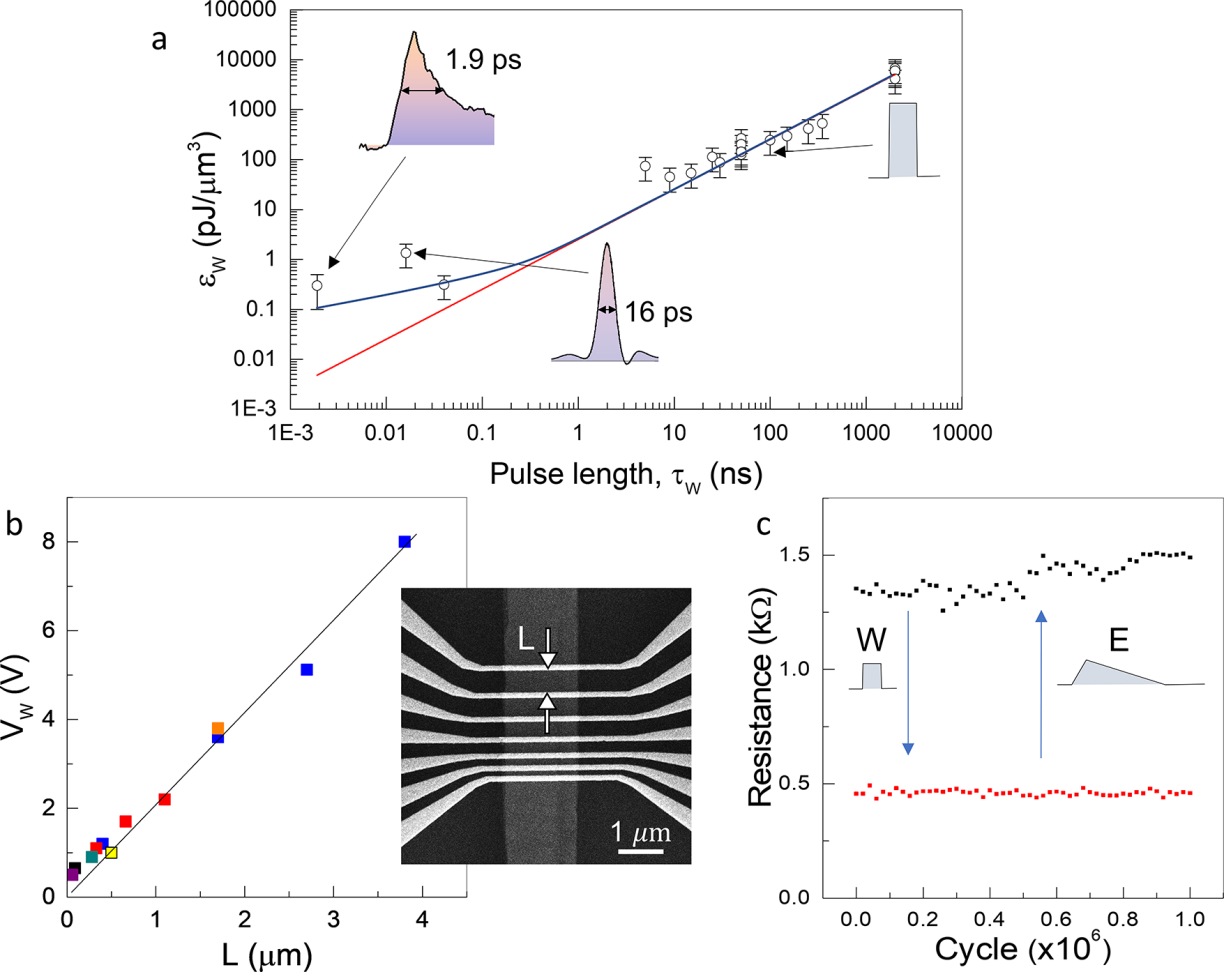
Speed and energy efficiency scaling at 20 K. (a) Switching energy density ε_W_ as a function of pulse length τ_W_. The inset shows the actual pulse shapes. Red line shows linear scaling, and blue line shows departure from linearity at short τ_W_. The data point at 1.9 ps was taken from ref (21). (b) Switching threshold voltage *V*_W_ as a function of distance between the electrodes *L*. The inset shows a device with variable *L* used in the measurement. Different symbol colors are for different physical devices. (c) Endurance measurement showing cycling between *R*_LO_ and *R*_HI_ for 10^6^ cycles. Each pair of points represents 2 × 10^4^ W/E cycles.

Using 60 GHz Au transmission line contacts, fabricated on ∼50 nm thick 1T-TaS_2_ flakes on Si/SiO_2_ substrates (see Figure 1a, Methods in Supporting Information), we systematically study the W sequence of multiple CCM devices for different W pulse lengths τ_W_ ranging from 16 ps to 20 μs generated by electronic pulse generators in a single-pulse mode. For each pulse length, the pulse voltage was incrementally increased until the switching threshold  was reached and recorded. Since the  depends on sample geometry, in Figure 3a, we plot the switching energy density instead:  ( is the crystal volume between electrodes, and  is defined as the switching energy). The error bars include the systematic error of measurements and the estimated variation between different devices. The red line shows a linear relation , with . At shorter pulse lengths  there is a departure from linearity denoted with the blue line, which most likely occurs due to an increase in transmission line losses in the GHz range. For the fastest device measured here (16 ps fwhm), the distance between contacts is  and threshold W voltage , which gives a switching energy per bit of  at 20 K. The resistance measured before and after switching is  and , respectively. The resistance ratio is lower than the value observed with longer W pulses at low temperatures such as that shown in Figure 2a, which may be attributed to incomplete switching. However, both  and  resistance states have long-term stability at this temperature (20 K). To explore the switching capabilities of the CCM device in the ultrafast limit, an electro-optical setup that allows for ps pulse generation and detection was reported in ref (21), from which we include a data point  = 1.9 ps in Figure 3a.

The scaling of the switching threshold voltage  with intercontact distance  is shown in Figure 3b. (A device with multiple *L* is shown in the inset.) Approximately linear scaling of  is observed over nearly 2 orders of magnitude, 60 nm < *L* < 4 μm, that extrapolates to the origin, with a slight departure from linearity at the smallest . Considering the fact that the EDS analyses do not show any intermediate layer between Au and 1T-TaS_2_ and that the work function mismatch suggests ohmic contact behavior, we cannot directly attribute the observed small deviation from linearity to electrical contacts. One possible reason for the departure at small sizes may be related to the fact that the device size approaches the intrinsic 10–20 nm domain size measured in the low-resistance state of single crystals.^9,20^ The efficiency of devices with feature sizes comparable to the domain size may be expected to decrease due to charge configuration pinning at the contacts.

In Figure 3c we show a typical endurance measurement at 20 K for a device with  nm. Square pulses with  and amplitude  V are used for W, and asymmetric triangular E pulses with peak amplitude  V and  are used for erase (shown in the inset to Figure 3c). Each pair of corresponding  and  values shown by black and red dots, respectively, represents a measurement after 20 000 W/E cycles. We see that  is remarkably stable over  cycles, while  initially increases slightly and later stabilizes after  cycles when the measurement was terminated.

An immediate application of the device could be in cryocomputing, which has been heralded as an obvious solution to the overall challenge of reducing dissipation of computer systems.^5,22^ In spite of huge research efforts and availability of superconducting circuits performing both single flux quantum (SFQ) and quantum information processing,^22^ the absence of a fast low-energy cryogenic memory has prevented significant upscaling,^4,5^ so CCM devices may offer a possible breakthrough. Considering the scaling limits of CCM devices, we find that while the measured value of  is small compared with other current memory devices, the observed scaling laws suggest that  can be reduced further by reducing  and/or . Optimization of microwave electrical contacts seems to be essential in reducing the  as well, since the losses in the transmission of GHz pulses are likely to be the cause for the departure from linear scaling in Figure 3a. Deviations from linearity might still be expected when  approaches the intrinsic switching time .^23^ These effects are likely to be important for device optimization, particulary for erase protocols.^7^ Fundamentally, the measured  is still significantly larger than the measured *microscopic* barrier *E*_B_ = 15–21 meV ((2.4–3.4) × 10^–6^ fJ) obtained from thermal activation measurements.^17^ This implies that in principle devices with much smaller  can be built before reaching fundamental limits. It is also instructive to compare  and  with the lowest possible energy difference between two states that can be discerned thermodynamically on the basis of their entropy difference  (the Brillouin–Landauer (BL) thermodynamic limit^24^). At  K,  fJ, so the presented CCM devices in Figure 3 are still far from this limit. For cryogenic memory applications, it is important that the CCM device could be driven by single flux quantum (SFQ) pulses. For a single SFQ pulse, the pulse energy may be estimated to  =  fJ, where we have assumed a typical critical current  μA in the SFQ driver circuit. For a realistic CCM device with  nm^2^ device area and a crystal thickness of 20 nm, the estimation from the linear fit in Figure 3a for switching with 2 ps^21^ pulses gives  fJ. Thus, on the basis of the presented scaling laws, CCM devices constructed using current fabrication techniques could be driven with single SFQ pulses, provided the coupling between the SFQ driver and the CCM microwave circuit is optimized.

In Figure 4 we compare operating parameters of the CCM with leading alternative technologies, including magnetic random-access memory (MRAM), phase change memory (PCM), and others. The smallest energy/bit values of 6 fJ/bit^25^ and 8 fJ/bit^26^ were reported for voltage-controlled magnetic anisotropy switching (MRAM) in Ta/CoFeB/MgO magnetic tunnel junctions (MTJ) and resistive switching in Ni/GeO_*x*_/HfON/TaN resistive random access memory (RRAM) devices, respectively (Figure 4b). The lowest theoretically predicted value for MTJs of a few  ^27^ is comparable to the , which was already experimentally achieved in CCM. This value is still much higher than the theoretical limit of  fJ predicted for CCM. PCMs have both significantly higher switching energies (>600 fJ ^28^) and switching voltages (>1 V ^29,30^) than CCM, and even the smallest memristors such as 6 nm crossbar memristor arrays have a write power of ,^31^ which is higher than the predicted ∼0.1  for a much bigger CCM device ( nm^3^), estimated from the linear fit in Figure 3a. We note that our demonstration of a man-made *nonvolatile* electronic memory device uses less energy per bit (2.2 fJ/bit) than a human brain (∼10 fJ/synapse) and potentially much less than artificial synapses.^32^ The CCM’s advantage is that it is >9 orders of magnitude faster.

**Figure 4:**
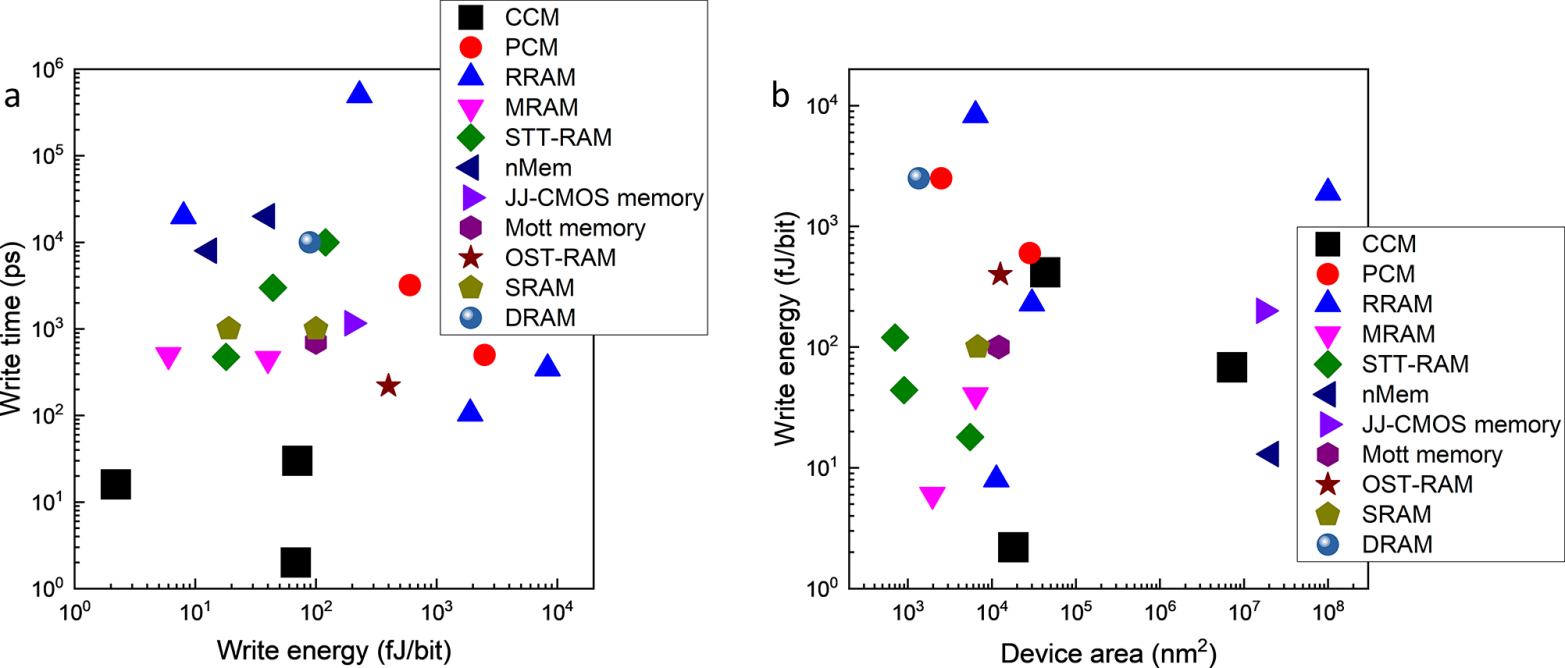
Measured switching energy *E*_W_ and speed of leading memory devices: (a) switching energy in correlation with device area; (b) switching times τ_W_ plotted against switching energy. References: PCM,^28,29^ RRAM,^26,33−35^ STT-RAM,^36−38^ MRAM,^25,39^ nMem,^40,41^ JJ-CMOS memory,^42^ OST-RAM,^43^ Mott memory,^44^ SRAM,^45−47^ and DRAM.^47^

Picosecond switching speeds of CCMs are similar to those reported by photomagnetic recording.^48^ However, the switching energy density per bit is significantly smaller for CCM: (0.3 J/cm^3^ for CCM^21^ vs 6 J/cm^3^ for photomagnetic recording), with the added advantage of two-contact electrical W/E and read (R) operations and the possibility of a very high packing density. For CCMs the current size limit is 10–20 nm, but for photomagnetic recording the size is limited by optical wavelengths used for W/E/R (hundreds of nm). Nanowire memory (nMem)^40,41^ based on superconductive loops and nanowire cryotrons, Josephson magnetic random access memory (JMRAM),^49^ superconducting quantum interference device (SQUID) memory,^50^ and hybrid Josephson complementary metal oxide semiconductor (JJ-CMOS) memory^42,51,52^ are all very promising solutions for a cryogenic memory. However, they are hard to scale down to the 10–20 nm regime and require additional driving periphery^4,53^ (nMem, JMRAM and SQUID memory) or require voltage amplification for proper operation^4^ (JJ-CMOS memory), which introduces additional dissipation into the circuits.

Thus, with presently demonstrated scalability (feature size, 60 nm to 4 μm; pulse length, 16 ps to 600 ms; voltage, 0.3–10 V) and with a wide operating temperature range (350 mK to 190 K), the CCM devices appear to be very versatile in comparison. The operating temperature range could be extended to higher *T* by appropriate choice of substrate.^17^ So far, no other electronically ordered material has been found to exhibit significant metastability and thus 1T-TaS_2_ currently is the only material for CCM application. The device variability is small, as seen from the data on multiple devices shown in the scaling plots. Importantly for applications, thin films of 1T-TaS_2_ can be grown by various means^54−56^ promising technological flexibility. For interfacing present CCM devices with single flux quantum devices, which is an obvious target application, nanocryotrons (nTrons) have been demonstrated to be a match in terms of output voltage, speed, and impedance.^57,58^ However, nTrons could introduce additional design complexity and dissipation due to higher bias currents,^51^ so direct driving by SFQ pulses might be preferable.

In comparison to complementary metal oxide semiconductor (CMOS) devices^42,51,52,59^ and nanowire memories,^40,41^ the CCM concept potentially offers advantages in terms of scaling, size, speed, energy efficiency, and operational simplicity. Compared with other fast memory, such as photomagnetic storage,^48^ CCMs are more energy efficient and offer much higher data packing densities. The disadvantage is low-temperature operation, but considering its primary virtues are speed and energy efficiency, the target applications are in the cryocomputing environment. The presently measured energy efficiency (2.2 fJ/bit) is a consequence of very short write pulses needed, allowed by the inherent switching mechanism. Thus, the values shown here are limited by device size and transmission characteristics of the high-speed microwave circuit, not the intrinsic mechanism.

We conclude that while 1T-TaS_2_ appears to be unique in exhibiting combined set of properties useful for CCM operation, multilayer structures may introduce additional functionality that may result in improved performance and extended temperature range of operation. The additional degrees of freedom arising from interlayer interactions and proximity coupling^60^ in 2D heterostructures may be expected to offer new possibilities for new CCM devices beyond the currently available materials.

## Supporting Information Available

Description of materials and fabrication methods; description of DC and fast electrical measurement (PDF)
