# Supplementary material for: Charge Configuration Memory Devices: Energy Efficiency and Switching Speed
Source: Nano Lett. 2022 Jun 10;22(12):4814–21. doi: 10.1021/acs.nanolett.2c01116 (PMC9228410; doi:10.1021/acs.nanolett.2c01116)
Supplement: Supplementary file 1 — nl2c01116_si_001.pdf [file nl2c01116_si_001.pdf]

Charge configuration memory devices – energy efficiency and switching speed

Anze Mraz*[1,4], Rok Venturini[1,3], Damjan Svetin[1,2], Vitomir Sever[1], Ian Aleksander Mihailovic[1], Igor Vaskivskyi[1], Bojan Ambrozic[2], Goran Dražić[9,10], Maria D'Antuono[6,7], Daniela Stornaiuolo[7,6], Francesco Tafuri[6,8], Dimitrios Kazazis[5], Jan Ravnik[5], Yasin Ekinci[5] and Dragan Mihailovic[1,2,3,9]

[1] Jozef Stefan Institute, Complex Matter Department F7, Jamova cesta 39, 1000 Ljubljana, Slovenia

[2] CENN Nanocenter, Jamova cesta 39, 1000 Ljubljana, Slovenia

[3]Faculty of Mathematics and Physics, University of Ljubljana, Jadranska cesta 19, 1000 Ljubljana, Slovenia

[4]Faculty of Electrical Engineering, University of Ljubljana, Tržaška cesta 25, 1000 Ljubljana, Slovenia

[5] Paul Scherrer Institute, Forschungsstrasse 111, 5232 Villigen PSI, Switzerland

[6] Dipartimento di Fisica "Ettore Pancini", Università di Napoli Federico II", Monte S. Angelo via Cinthia, 80126 Napoli, Italy

[7]CNR-SPIN, Complesso Monte Sant'Angelo via Cinthia, 80126 Napoli, Italy

[8]CNR - Istituto Nazionale di Ottica (CNR-INO), Largo Enrico Fermi 6, 50125 Florence, Italy

[9]Jozef Stefan International Postgraduate School, Jamova cesta 39, 1000 Ljubljana, Slovenia

[10]National Institute of Chemistry, Dept. of Materials Chemistry, Hajdrihova 19, 1000 Ljubljana, Slovenia

*corresponding author's email: anze.mraz@ijs.si

Methods. The 1T-TaS_2 single crystals were synthesized using the vapor phase transport method. More than 1000 CCM devices (such as shown in Fig. 1a and the insert to Fig. 3b) were fabricated by deposition of typically $30 - 100$ nm thick 1T-TaS_2 crystals on Si/SiO_2 substrates. 80 nm thick sputtered Au electrodes with a 5 nm Au/Pd contact layer were then deposited using e-beam or laser lithography. Performing the fabrication procedure in an inert atmosphere (glove box with nitrogen) and standardization of lithographic processes have increased the yield for CCM devices above 90% and have resulted in very high-quality electrical contacts. The contact resistance with PdAu/Au electrodes, obtained from a 4-probe measurement was typically $100 - 200\ \Omega$. DC transport device measurements were performed using a standard four-point contact technique. For $\tau_W > 4$ ns an electrical signal generator (Siglent SDG 1050) was used, while for $\tau_W < 100$ ps electrical pulses were generated using an amplified SYMPULS signal generator giving ± 2.2 V at $\tau = 16$ ps (FWHM, 11 ps rise-time at the input to the cryostat after the amplifier). For frequencies > 1 GHz, transmission lines were used throughout, such as those shown in Fig. 1a.
